# Genetic Characterizations and Molecular Evolution of VP7 Gene in Human Group A Rotavirus G1

**DOI:** 10.3390/v12080831

**Published:** 2020-07-30

**Authors:** Nan Zhou, Lu Zhou, Bei Wang

**Affiliations:** 1 Key Laboratory of Environmental Medicine and Engineering of Ministry of Education, Department of Epidemiology and Statistics, School of Public Health, Southeast University, Nanjing 210009, China; sduzhounan@163.com; 2 Department of Acute Infectious Diseases, Jiangsu Provincial Center for Disease Control and Prevention, Nanjing 210009, China; zhoulu1212@163.com

**Keywords:** human rotavirus, G1 genotype, VP7 gene, evolution

## Abstract

Rotavirus group A (RVA) G1 is one leading genotype circulating in humans worldwide, and related molecular information from a global perspective is still limited. Here, we present a comprehensive description of the genetic characterizations and molecular evolution of the RVA G1 VP7 gene. Our results show that RVA G1 can be divided into two lineages and multiple sub-lineages with a relatively high genetic diversity. Vaccine strains are phylogenetic, closer to lineage I. The evolutionary rate of the RVA G1 VP7 gene is 8.869 × 10^−4^ substitutions/site/year, and its most recent common ancestor was in 1933. The RVA G1 VP7 gene shows a linear evolution at the nucleotide level and a linear accumulation of difference at the amino acid level. Sub-lineage replacement of G1 VP7 gene is also observed and the effective population size of the G1 VP7 gene has had great change in the past decades and has remained stable in recent years. Altogether, the RVA G1 VP7 gene constantly evolves and there is no clear evidence that the evolution of the RVA G1 VP7 gene was influenced by vaccines. Continuous surveillance is still indispensable to evaluate the molecular epidemiology of RVA, especially in the post-vaccination era.

## 1. Introduction

Rotavirus group A (RVA) is a leading viral agent of acute gastroenteritis (AGE) in human populations. There were more than 120,000 deaths and 250,000,000 episodes of diarrhea among children < 5 years old attributed to RVA in 2016 worldwide [1]. RVA belongs to *Reoviridae* family and possess a genome with 11 double-stranded RNA (dsRNA) segments, encoding six structural proteins (VPs) and six nonstructural proteins (NSPs) [2,3]. RVA strain is classified by a binary genotype system based on the VP7 (glycoprotein, G genotype) and VP4 (protease-sensitive protein, P genotype) proteins, and more than 35 different glycoprotein (G) genotypes (VP7 gene) and 50 different protease (P) genotypes (VP4 gene) have been recognized [4]. Of these, G1–G4, G9, G12, P[4], P[6], P[8] are the predominant genotypes worldwide [5].

To date, two live attenuated rotavirus vaccines (RotaTeq and Rotarix) have been recommended by the WHO since 2009, and have been introduced in 109 countries so far in order to reduce the disease burden of RVA [6]. Rotarix is a monovalent vaccine and contains a single attenuated human G1P[8] strain [7]. RotaTeq is a pentavalent human-bovine (WC3) reassortant rotavirus vaccine and combines five separate reassortant strains (G1–G4, P[8]) [8]. RVA-associated gastroenteritis in hospitalizations and outpatient visits has showed a decline due to the introduction of universal mass vaccination around the world. Through ongoing epidemiological surveillance, the impact of RVA vaccines on genotype diversity has been evaluated in some regions [4,9], and increased diversity and differences in genotype dominance have been observed, such as the emerging equine-like G3P[8] strain [10,11].

Nevertheless, at present, little is known about the molecular evolution of specific RVA genotypes. The two licensed vaccines both include the G1 genotype, and G1 still remains a largely dominant G genotype worldwide [12,13,14]. It was also reported that G1 was the predominant shedding vaccine-related viral particle in vaccines [15,16], which may have an effect on the evolution of wild-type G1 circulating in populations. Thus, research on the long-term evolutionary characterizations of RVA G1 from a global perspective is desirable and is currently inadequate. Here, we used the complete genome of RVA G1 VP7 strains in the GenBank database and conducted a comprehensive description to expand our understanding of the genetic evolution of the RVA G1 VP7 gene.

## 2. Materials and Methods

### 2.1. Strain Selection

The complete genome (981 nt) of the RVA G1 VP7 gene was retrieved from NCBI’s GenBank Database (accessed on 29 February 2020). Ambiguous strains with an unknown collection year and undetermined nucleotides (such as R, Y, and S) were excluded. Strains associated with vaccines, cultivated in eukaryotic cells, from environmental samples, and presenting recombination signals determined by Recombination Detection Program (RDP) v4.56 software were also excluded [17]. In order to reduce the number of strains in the dataset, one strain was randomly selected from a group of homologous sequences with the same collection year according to an identity ≥99.6%.

### 2.2. Genetic Diversity of RVA G1 VP7 Gene

Alignment of included strains was conducted by BioEdit 7.1.3.0, which was also used to calculate the nucleotide and amino acid identities [18]. Then, the IQ-TREE web server was used to estimate the best-fit nucleotide substitution model on the basis of Bayesian Information Criterion (BIC) scores [19], and a phylogenetic tree was constructed by MEGA v7.0.26 based on the maximum likelihood (ML) method with 1000 bootstrap replications for branch support [20]. Pairwise nucleotide p-distance and the inter-lineage mean amino acid distance were also estimated by MEGA v7.0.26. Shannon entropy values for each amino acid position were calculated by the web serve of Shannon Entropy-Two tool (www.hiv.lanl.gov) to determine if there was greater variability between different lineages, and Phylo-mLogo-2.3 was used to visualize the amino acid difference between lineages [21].

### 2.3. Selection Pressure Analysis

Selection pressure analysis on the RVA G1 VP7 gene was carried out to estimate the nonsynonymous (dN) and synonymous (dS) substitution ratio (dN/dS) using the online Datamonkey server [22]. Single-likelihood ancestor counting (SLAC) and fixed effects likelihood (FEL) methods were used to determine the site under positive selection (dN > dS) or negative selection (dN < dS). Mixed effects model of evolution (MEME) method was used to determine the site under positive selection. The *p*-value threshold was 0.05.

### 2.4. Root-to-Tip Analysis

To reconstruct a relationship between genetic divergence and time, TempEst v1.5 was used to infer the root-to-tip divergence based on the ML phylogenetic tree for each lineage [23]. Then, the root-to-tip divergence against the collection time for each strain was plotted and a locally weighted scatterplot smoothing (LOWESS) fit line was added to evaluate the fluctuation of evolution over time. Linear regression analysis was also performed to demonstrate the linear trend of evolution.

### 2.5. Accumulation Pattern of Amino Acid Substitutions

To discuss the accumulation pattern of amino acid substitutions of the RVA G1 VP7 gene, an algorithm was used as previously described [24]. Briefly, pairwise amino acid differences among strains within the same lineage were calculated by MEGA v7.0.26 and averaged based on the timespan. Then, the mean pairwise amino acid difference was plotted against the timespan of isolation. Linear regression was also estimated to evaluate the possible linear accumulation trend and the accumulative rate.

### 2.6. Evolutionary Dynamics Analysis

Bayesian Markov Chain Monte Carlo (MCMC) method in BEAST package v1.8.3. was used to carry out evolutionary analysis of the RVA G1 VP7 gene [25]. The best-fit nucleotide substitution model (HKY + G) was inferred as mentioned above. Strict clock model and tree prior of Bayesian skyline coalescent were selected to estimate the evolutionary dynamic parameters including evolutionary rate (nucleotide substitutions/site/year), time to the most recent common ancestor (TMRCA) and Bayesian skyline plot, which were summarized in Tracer v1.6 (http://tree.bio.ed.ac.uk/software/tracer/) with effective sample sizes (ESS) >200 when MCMC runs were carried out for 100 million. A maximum clade credibility (MCC) time-dated tree was generated after 10% burn-in by TreeAnnotator v1.8.2 (http://tree.bio.ed.ac.uk/software/) and visualized by FigTree v1.4 [26].

## 3. Results

### 3.1. Genetic Characterizations of RVA G1 VP7 Gene

**Figure 1:**
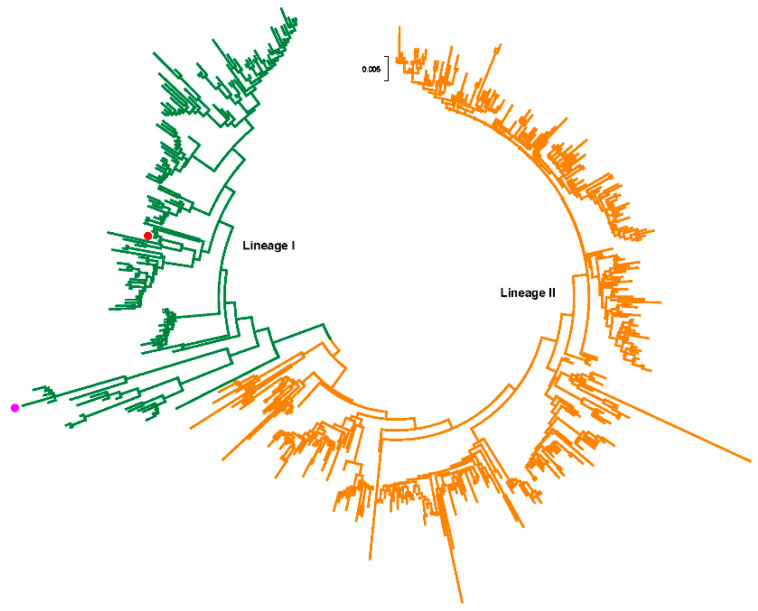
Phylogenetic relationship of RVA G1 strains based on the complete genome of the VP7 gene using maximum likelihood method. Red and light purple dots indicate Rotarix and RotaTeq vaccine reference strains, respectively.

According to inclusion and exclusion criteria, a total of 544 strains were analyzed in this study (Supplemental Table S1). These strains were collected from 1974 to 2018. The nucleotide and amino acid identities ranged from 88.9% to 100% and 88.3% to 100%, respectively. We then constructed a ML phylogeny based on the complete genome of the RVA G1 VP7 gene. RVA/Human-TC/USA/Rotarix/2009/G1P[8] (accession number: JX943614) and RVA/Vaccine/USA/RotaTeq-WI79-9/1992/G1P7[5] (accession number: GU565057) served as two vaccine reference strains. Our results showed that G1 VP7 gene can be divided into two lineages (Figure 1) and vaccine strains were all clustered into lineage I.

Excluding vaccine strains, the strains of lineage I isolated from 1974 to 2016 with the nucleotide and amino acid identities ranged from 91.8% to 100% and 92.3% to 100%, respectively. The strains of lineage II isolated from 1987 to 2018 with the nucleotide and amino acid identities ranged from 91.7% to 100% and 90.1% to 100%, respectively. The geographical and temporal distributions of divergence between lineage I and II strains were observed (Supplemental Table S2). Lineage II circulated more extensively than lineage I, and most of the countries that only had lineage II circulating were from Africa where RVA vaccines have been implemented in these regions. In most countries, two lineages circulated and the circulation timespans of lineage I and II were similar. The mean numbers of amino acid difference within lineage I and II were 7.737 ± 1.231 and 7.352 ± 1.187, respectively. The mean number of amino acid difference between lineage I and II was 18.907 ± 3.315.

**Figure 2:**
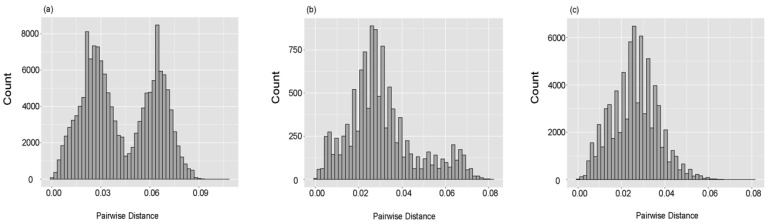
Distribution of nucleotide pairwise distances of RVA G1 VP7 strains: (**a**) all analyzed strains, (**b**) lineage I, (**c**) lineage II.

Pairwise p-distances among all analyzed strains were also calculated and ranged from 0 to 0.107 with a mean value of 0.042. The histogram presented an obvious bimodal distribution, revealing the form of genetically distant two lineages (Figure 2a). For each lineage, the normality of the distribution of pairwise distance was tested using the Kolmogorov–Smirnov method, and the *p*-values of lineage I and II were both less than 0.001, indicating they did not meet the normal distribution. The histograms of pairwise distances showed that lineage I exhibited an atypical unimodal distribution and had a main peak and a minor uplift (Figure 2b), and lineage II displayed a unimodal distribution (Figure 2c). Pairwise distances of strains in lineage I ranged from 0 to 0.082 with a mean value of 0.031, and pairwise distances of strains in lineage II ranged from 0 to 0.081 with a mean value of 0.026.

**Figure 3:**
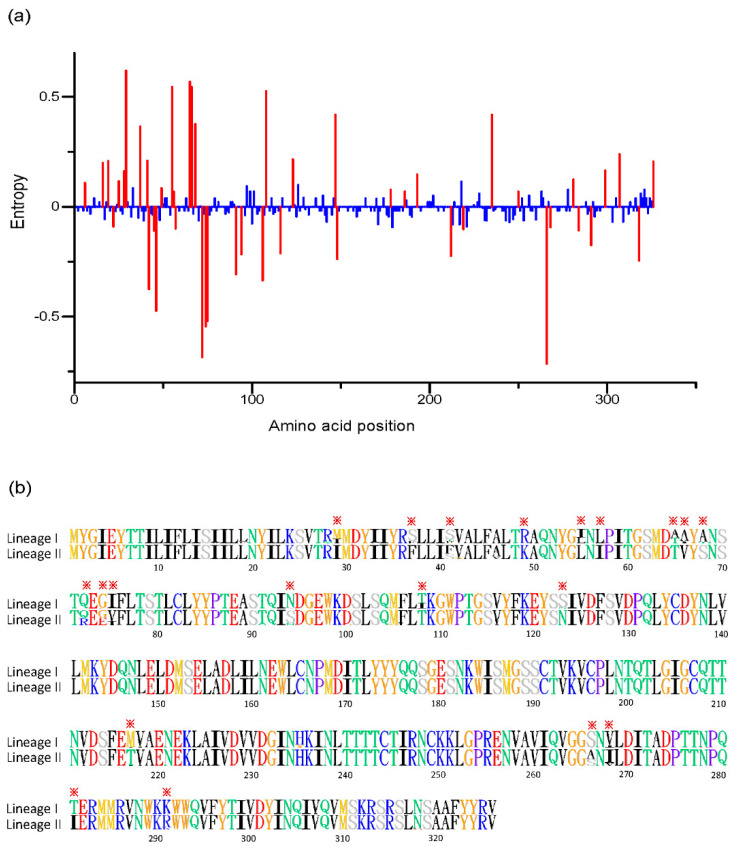
(**a**) Shannon entropy comparison between two lineages with significant differences colored in red. (**b**) Amino acid alignment between two lineages with significant specific amino acid residues labeled with asterisks.

Entropy values for each amino acid position were compared to determine if there is greater variability between two lineages, and 46 positions (at positions: 6, 16, 19, 22, 25, 28, 29, 37, 41, 42, 45, 46, 49, 55, 56, 57, 65, 66, 68, 72, 74, 75, 91, 94, 106, 108, 116, 123, 147, 148, 178, 186, 193, 212, 219, 235, 250, 266, 268, 281, 284, 291, 299, 307, 318, 326) were identified with significant difference (Figure 3a). Most of these were located in the region with amino acid position 1~100. Then, alignment of amino acids of the RVA G1 VP7 gene was conducted and visualized to explore the specific amino acid residues for each lineage. Our results showed (Figure 3b) that there were about 20 sites showing specific differences in the amino acid residues between two lineages (at positions: 29, 37, 41, 49, 55, 56, 65, 66, 68, 72, 74, 75, 94, 108, 123, 217, 266, 268, 281, 291), providing us with two distinct identification codes for lineage. The code of M(I)SSRI(L)LA(T)A(V)AQGINT(I)S(N)MSV(I)TK was for lineage I, and lineage II had an identification code of IFF(S)KLITVSQ(R)G(E)V(I)STNTA(S)I(V)IR. In addition, comparison of G1 VP7 antigenic epitopes (7–1a, 7–1b and 7–2 epitopes) between vaccine strains and circulating lineages showed that lineage II displayed a different amino acid residue at position 94, 123, 217, 291 with vaccine strains (Table 1).

Selection pressure analysis was also performed on the RVA G1 VP7 gene. For lineage I, a total of 38 and 82 negative sites were identified by the SLAC and FEL methods, respectively. Five sites (at positions: 14, 19, 23, 323, 326) under positive selection were observed by the MEME method. For lineage II, a total of 82 and 122 negative sites were identified by the SLAC and FEL methods, respectively. The SLAC method also identified one site (at position 212) under positive selection pressure. By the MEME method, ten sites (at positions: 23, 24, 42, 185, 212, 218, 281, 291, 309, 323) under positive selection were observed.

### 3.2. Root-to-Tip Divergence Analysis

**Figure 4:**
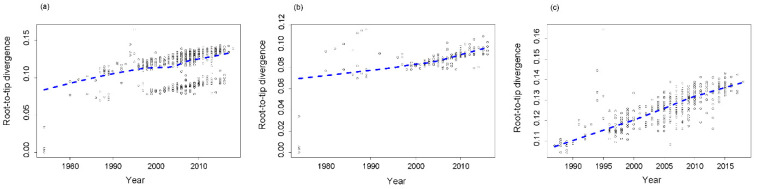
Root-to-tip divergence plots of the RVA G1 VP7 gene: (**a**) all analyzed strains, (**b**) lineage I, (**c**) lineage II. The *Y*-axis indicates the root-to-tip divergence based on the maximum likelihood tree, and the *X*-axis indicates the collection year. Each strain is represented by a circle, and the dashed line indicates a locally weighted scatterplot smoothing (LOWESS) line.

In order to evaluate time-clock signal in the evolution of RVA G1 VP7 gene, the plots of root-to-tip divergence against the time of sampling of each strain were constructed and LOWESS fit-line was added. Our results showed that there were two clusters observed in the plot of all analyzed strains, and LOWESS fit-line indicated that there was a slight decline in the evolutionary rate of the G1 VP7 gene around 2000 (Figure 4a). LOWESS fit-line of lineage I showed that there was a slight rise in the evolutionary rate around 2000 (Figure 4b) and the LOWESS fit-line of lineage II was almost linear (Figure 4c). Linear regression analysis indicated that lineage I and II all exhibited a positive correlation between genetic divergence and sampling time, and both evolved with a moderate linear trend (the values of R^2^ were 0.366 and 0.536 for lineage I and II, respectively), which also meant that RVA G1 VP7 gene was suitable for phylogenetic molecular clock analysis in BEAST program. Besides, the slopes between lineage I and II were similar (1.028 × 10^−3^ vs. 9.496 × 10^−4^).

### 3.3. Accumulation Pattern of Amino Acid Substitutions

**Figure 5:**
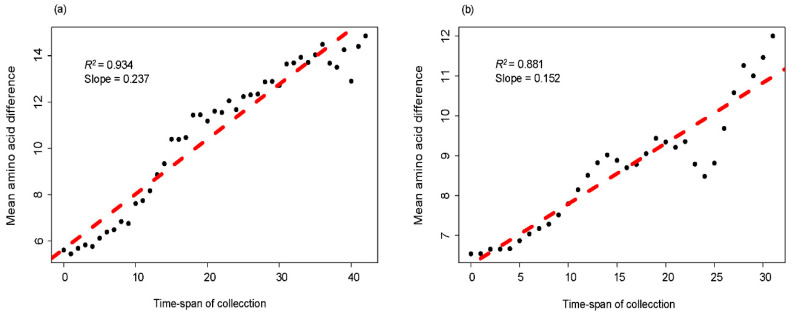
Accumulation plots of amino acid over time of the RVA G1 VP7 gene: (**a**) Lineage I, (**b**) lineage II. The *X*-axis indicates the timespan of collection, and the *Y*-axis shows the mean amino acid difference. The dashed line indicates a linear regression line.

In order to discuss the relationship between amino acid difference and timespan among strains at a lineage level, mean pairwise amino acid difference was plotted against the timespan of collection. Our results showed that the mean amino acid difference of the RVA G1 VP7 gene accumulated continually over time both in lineage I and II (Figure 5a,b). Linear regression analysis revealed that two lineages all presented strong linear accumulation, and the values of R^2^ were 0.934 and 0.881, respectively. Furthermore, lineage I had a faster accumulation rate (slope = 0.237) than lineage II (slope = 0.152).

### 3.4. Time-Scaled Evolutionary Analysis

**Figure 6:**
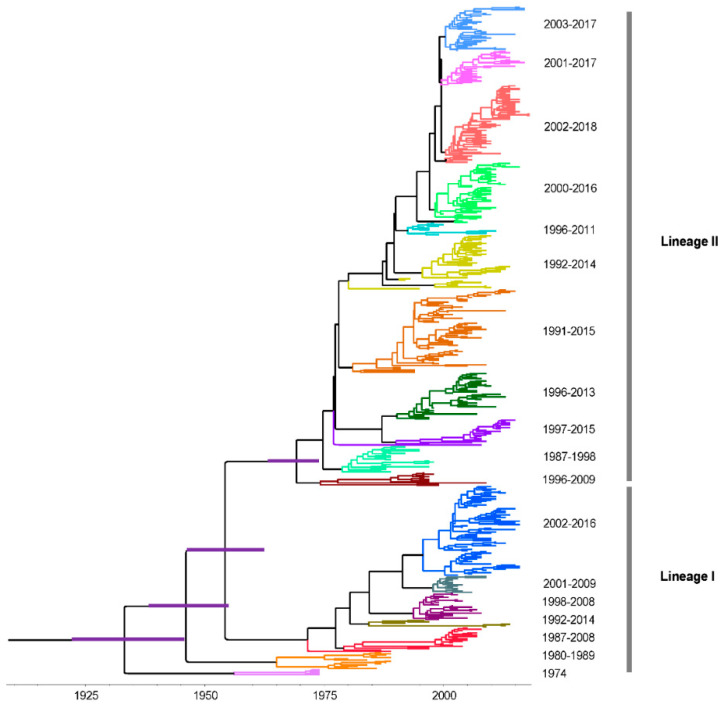
Time-scaled phylogenetic tree of RVA G1 VP7 gene by using the Bayesian Markov Chain Monte Carlo method. Branches are scaled in time and strains are colored according to sub-lineages. Node bars indicate 95% highest posterior densities of the most recent common ancestor.

In order to discuss the evolutionary dynamic of G1 strains, time-scaled MCC tree was constructed by a Bayesian MCMC method and the same two lineages were observed when compared with ML tree (Figure 6). Multiple sub-lineages were also identified at lineage level and lineage II had more number. Additionally, a ladder-like phylogenetic topology was observed in lineage I, which was not obvious in lineage II. The timespans of sub-lineages in lineage II were longer than lineage I, which were more than 10 years.

The overall evolutionary rate of RVA G1 VP7 gene was estimated to be 8.869 × 10^−4^ substitutions/site/year (95% highest posterior densities [HPDs]: 8.024 × 10^−4^–9.802 × 10^−4^ substitutions/site/year). For lineage I and II, the evolutionary rates were 8.023 × 10^−4^ substitutions/site/year (95% HPDs: 6.699 × 10^−4^–9.400 × 10^−4^ substitutions/site/year) and 8.796 × 10^−4^ substitutions/site/year (95% HPDs: 7.754 × 10^−4^–9.812 × 10^−4^ substitutions/site/year), respectively. The most recent common ancestor (TMRCA) of this MCC tree was found around 1933 (95% HPDs: 1922~1946), and the divergence times of lineage I and II were 1936 (95% HPDs: 1922–1946) and 1954 (95% HPDs: 1943–1964), respectively.

### 3.5. Phylodynamics of RVA G1 VP7 Gene

**Figure 7:**
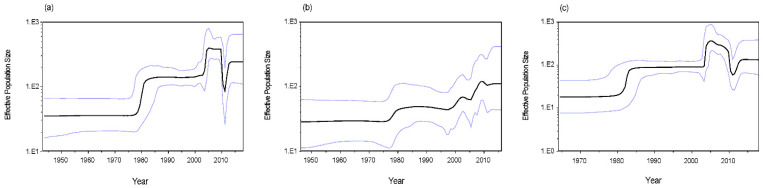
Bayesian skyline plots of RVA G1 VP7 gene: (**a**) all analyzed strains, (**b**) lineage I, (**c**) lineage II. The *Y*-axis indicates the effective population size. Mean effective population is shown as a black line. The 95% highest posterior densities are shown as light blue lines.

Bayesian skyline plots were constructed to assess the change in the effective population size of the RVA G1 VP7 gene. The mean effective population size of the whole G1 strain started to increase around 1980 and then remained stable. After 2003, it begun to increase abruptly and showed a sharp fall in 2010 (Figure 7a). The differences in the Bayesian skyline plots between lineage I and II were considerable. For lineage I, the mean effective population size presented an increasing trend since 1975 (Figure 7b). For lineage II, the mean effective population size was similar to the change of the whole G1 strains (Figure 7c). From 2005 to 2010, the effective population size of lineage I had a growing trend, but lineage II showed a declining trend.

## 4. Discussion

RVA G1 is a prominent G genotype circulating in human populations. In this study, we presented a comprehensive bioinformatics description of the molecular evolution of the RVA G1 VP7 gene using a large number of strains from a public database. Our results revealed that the intra-genotypic diversity of the RVA G1 VP7 gene was relatively high, and the nucleotide and amino acid identities are similar to other gastroenteritis viruses, such as some classic human astrovirus serotypes [24]. The phylogenetic relationship of the RVA G1 VP7 gene was also evaluated through the construction of an ML tree, and we revealed two lineages which are consistent with Novikova’s and Fujii’s results [27,28]. At present, there is no clear criterion to define a lineage of the RVA G1 VP7 gene and many studies have identified a different number of genetic lineages [14,29,30]. Previous research by Phan et al. recognized more than 10 lineages [31]. However, we think that the classification of two lineages in this study is appropriate, because the histogram of pairwise nucleotide distances displayed an obvious bimodal distribution and two clusters were also observed in the root-to-tip divergence analysis, providing us with more evidence on the form of two genetically distant lineages. The histogram of pairwise nucleotide distances of lineage I was an atypical unimodal distribution which may harbor two main sub-lineages in lineage I, including Rotarix and RotaTeq vaccine strains, respectively. However, the lower number of sequences in the latter gives the potentially false indication of a unimodal distribution.

The VP7 glycoprotein gene of RVA encodes 326 amino acid residues and consists of nine variable regions (VR1-VR9) among the various G genotypes. Four of these divergent regions, VR5 (region A: amino acid 87–101), VR7 (region B: amino acid 141–151), VR8 (region C: amino acid 208–224) and VR9 (region F: amino acid 235–242), are regarded as the major antigen sites [32,33,34]. In this study, the amino acid differences between lineages were explored. There was an attempt to identify the differentiation marker between lineages and about 20 specific sites were recognized, which are mostly located at the forepart of VP7 gene and little mapped to four divergent regions. Only a few significant specific amino acids were located in hydrophilic regions, according to previous reported hydrophobicity profiles [35], indicating that most of them may fall within the internal structure. However, this is not surprising because the aforementioned divergent and hydrophilic regions were identified at the genotype level. At the lineage level within one G genotype, the variation of antigen sites might be different from that. Nevertheless, some variable sites at two structurally defined antigenic epitopes (7–1a, 7–1b and 7–2 regions) are noteworthy, which are crucial for virus recognition by antibodies [36,37,38]. In this study, there were four observed substitutions at positions 94 (N→S), 123 (S→N), 217 (M→T) and 291 (K→R) located in that area. More importantly, lineage I shared the same amino acid residues with vaccine strains at these four sites, but lineage II showed a discrepancy. Considering that lineage I and vaccine strains owned a closer phylogenetic relationship, licensed RVA vaccines may result in a stronger protective effect on circulating strains of lineage I than lineage II. In addition, selection pressure analysis indicated that lineage I displayed fewer sites under positive selection. It might be that lineage II was circulating more extensively in regions where the vaccines have been implemented early and would therefore have been under immune pressure for a longer period. That may explain the larger number of sites under positive selection and the amino acid divergence of lineage II from the vaccines’ strains.

On the whole, there were more observed divergent sites between lineages in this study than previous research [31]. More sites may indicate a more precise distinction of lineages, but there was less repetition between these specific sites. Although our results may be more accurate due to the large number of included strains for alignment, reconsideration of the signature code for each lineage is still needed. Escape from neutralizing antibody due to the key amino acid substitutions in VP7 epitopes among circulating strains is possible, and there are still no available data on vaccine efficacy against intra-genotypic lineages. Thus, research on the antigenic relationships between different G1 lineages should be conducted in the future.

Root-to-tip divergence plot is often used to analyze the temporal structure of strains [23]. In this study, we used it to explore the evolutionary fluctuation of the RVA G1 VP7 gene over sampling time by the LOWESS fit-lines, which were relatively stable, revealing that there was no obvious change during the evolution of the RVA G1 VP7 gene. The subtle change around 2000 may be attributed to natural fluctuation but not pressure from the vaccine, because the vaccine against RVA was not licensed at that time. The accumulation pattern of amino acid substitutions has been used to determine the evolutionary patterns of viruses [24,39], which may be a signal to evaluate the relative prevalence of genotypes (or serotypes) in some viruses. We found that the amino acid accumulation of the RVA G1 VP7 gene presented a strong linear relationship at the lineage level, indicating an evolving but not static pattern. This evolving pattern is in accordance with the higher prevalence of RVA G1 in the populations. In addition, lineage I had a faster amino acid accumulation rate, but still needs more epidemiology information of different lineages to determine whether a faster accumulation rate means a higher prevalence at the lineage level.

Lineage replacement is considered to be an important evolutionary mechanism for RVA to adapt to different immunological environments [40,41,42,43,44]. However, this replacement was not obtained in this study, and in most of countries where lineage I and II circulated, the timespan of the two lineages we identified were similar and presented a lot of overlap, indicating that they co-circulated for a long time in the populations. Only represented strains were selected for the analysis because of the limitations in the software’s capacity, and the geographical and temporal information in this work may be limited. The spatial–temporal replacement of lineages of RVA should be continuously assessed. Interestingly, sub-lineage replacement of the RVA G1 VP7 gene was observed in the MCC tree, especially in lineage I. This may help to avoid the influence of immune pressure and contribute to the continuous circulation of RVA strains at lineage level. Furthermore, we speculate that the RVA G1 VP7 gene may evolve at the sub-lineage level. The TMRCA of RVA G1 VP7 gene has been reported to be the 1950s and 1980s [44,45], but these studies were regional. Our result displayed that the TMRCA of the RVA G1 VP7 gene can date back to the 1930s on the global scale. The estimation of the evolutionary rate of the G1 VP7 gene in this study was relatively higher (close to 10^−3^ substitutions/site/year) and similar to previously reported data [28,44,45]. Substitution rates are deemed to be linked to the rates of inter-host transmission [46]. The higher evolutionary rate may partially explain the higher prevalence of RVA G1 in the humans. The slope of the root-to-tip divergence plot also denotes the evolutionary rate of specific strains, more importantly, which can be used to evaluate the fluctuation of evolution by analyzing the change of slope. In this view, our results indicate that the evolutionary rate of the G1 VP7 gene has been relatively stable over the past decades.

The analysis of Bayesian skyline plot is often used to explore the relative effective population size of viruses over time [47]. In this study, we found that the effective population size of the RVA G1 VP7 gene exhibited relatively large changes, and the Bayesian skyline plots of lineage II and the whole G1 strains were similar since the number of strains belonging to lineage II were in the majority of the whole analyzed strains. However, the reason for the distinct differences in the Bayesian skyline plots between lineage I and II is to be determined. Furthermore, the stable Bayesian skyline plots in recent years may predict that the circulation of RVA G1 in humans will remain steady for some years.

In conclusion, the spatial–temporal distribution of analyzed sequences was not uniform, and over-representation of samples in this work is possible. Thus, some results we reported in this work may be closer to specific geographic areas and years, which frequently had high quality and a great extent of surveillance data and outbreak-associated epidemiologic surveys. Although selection bias may affect our results, we believe that our results are of great value. We found that the RVA G1 VP7 gene can be divided into two lineages and multiple sub-lineages with a relatively higher genetic diversity. Vaccine strains are phylogenetic, closer to lineage I. The evolutionary rate of the VP7 gene is high. In addition, the G1 VP7 gene presents a linear evolution at the nucleotide level and displays a linear accumulation of difference at the amino acid level within two lineages. Sub-lineage replacement may contribute to the constant circulation of RVA G1 in humans. Some differences in the effective population size between two lineages were also observed. Overall, there is no clear evidence that the evolution of RVA G1 VP7 gene was influenced by vaccines. In the future, constant surveillance for RVA should continue to elucidate the effect of vaccines on the prevalent genotypes and evolutionary characterizations of RVA.

## Figures and Tables

**Table 1:** Comparison of RVA G1 VP7 antigenic epitopes between vaccine strains and circulating lineages.

Position	7–1a														7–1b							7–2							
	87	91	94	96	97	98	99	100	104	123	125	129	130	291	201	211	212	213	238	242	145		146	147	148	190	217	221	264
RotaRix	T	T	N	G	E	W	K	D	Q	S	V	V	D	K	Q	N	V	D	N	T	D		Q	N	L	S	M	N	G
RotaTeq	T	T	N	G	D	W	K	D	Q	S	V	V	D	K	Q	N	V	D	N	T	D		Q	S	L	S	M	N	G
Lineage I	T	T	N	G	E	W	K	D	Q	S	V	V	D	K	Q	N	V	D	N	T	D		Q	N	L	S	M	N	G
Lineage II	T	T	S	G	E	W	K	D	Q	N	V	V	D	R	Q	N	V	D	N	T	D		Q	N	L	S	T	N	G

The different amino acid residues in lineage II are in red compared with lineage I and vaccine strains.

## Supplementary Materials

The following are available online at https://www.mdpi.com/1999-4915/12/8/831/s1, Table S1: RVA G1 VP7 strains analyzed in this study. Table S2: Geographical and temporal information of strains in lineage I and II.
