# Supplementary material for: Genetic Characterizations and Molecular Evolution of VP7 Gene in Human Group A Rotavirus G1
Source: Viruses. 2020 Jul 30;12(8):831. doi: 10.3390/v12080831 (PMC7472278; doi:10.3390/v12080831)
Supplement: Supplementary file 1 [file viruses-12-00831-s001.pdf]

Table S1 RVA G1 VP7 strains analyzed in this study

Accession number	Country	Collection date
JX406755	USA	1974
KT694944	USA	1974
KT694966	USA	1974
KT694988	USA	1974
LC438384	JPN	1974
JX458953	ARG	1980
JQ710662	ARG	1980
JQ710663	ARG	1982
JQ710664	ARG	1984
JX458954	ARG	1984
JX458955	ARG	1986
JQ710665	ARG	1986
AB081794	JPN	1987
JQ710666	ARG	1987
KM977723	RUS	1987
KT694999	USA	1988
AB081795	JPN	1988
AB081796	JPN	1989
KT695054	USA	1989
JX458956	ARG	1989
JQ710667	ARG	1989
AF043678	AUS	1992
AF480296	URY	1996
JQ710669	ARG	1997
JX458958	ARG	1997
JX458957	ARG	1997
JF968560	BRA	1998
JF968561	BRA	1999
JN232053	BRA	1999
JF968563	BRA	1999
JN232050	BRA	1999
JN232065	BRA	2000
JX458963	ARG	2000
KT248550	BEL	2000
JN258813	BEL	2000
KJ753253	ZAF	2001
JF968568	BRA	2001
JN232068	BRA	2002
KJ753517	ZAF	2002
EF179187	PRY	2002
KJ559047	PRY	2002

JN258846	BEL	2002
KP752554	ZAF	2002
KJ751795	ZAF	2002
JX458965	ARG	2002
JN258857	BEL	2002
KP752431	ZAF	2002
JN258874	BEL	2002
EF179189	PRY	2003
KJ752808	ZAF	2003
JF968574	BRA	2003
JN258895	BEL	2003
KJ626767	PRY	2003
JF968573	BRA	2003
KJ752524	ZAF	2003
KJ753846	ZAF	2003
GU377134	RUS	2003
GU377133	RUS	2003
JF490154	AUS	2004
JF968576	BRA	2004
JF968580	BRA	2004
JF490539	AUS	2004
AB585913	CHN	2004
GU377136	RUS	2004
KP752747	ZAF	2004
GU377135	RUS	2004
JF490549	AUS	2005
GQ117001	RUS	2005
JF968583	BRA	2005
JX470504	CA	2005
JF968582	BRA	2005
JF490836	USA	2005
JF490330	AUS	2005
HQ392040	BEL	2005
JF490747	USA	2005
JF490935	USA	2006
HQ392150	BEL	2006
JF490506	AUS	2006
FJ435208	RUS	2006
GQ117002	RUS	2006
FJ948854	IND	2006
HQ392074	BEL	2006
JF490946	USA	2006
HM773851	USA	2007
HM773774	USA	2007

JQ043275	THA	2007
JX841119	RUS	2007
JF490406	AUS	2007
JQ043277	THA	2007
JN192099	IND	2007
HM773818	USA	2007
JQ043278	THA	2007
JF490396	AUS	2007
GQ452913	RUS	2007
HQ392371	BEL	2008
JX470512	CA	2008
GQ452921	RUS	2008
HQ392261	BEL	2008
HQ392250	BEL	2008
HQ738620	RUS	2008
HQ738628	RUS	2008
HQ738627	RUS	2008
JN706198	THA	2009
HQ392415	BEL	2009
HQ392406	BEL	2009
KX638538	IND	2009
GQ996836	THA	2009
KJ752031	ETH	2009
GQ452914	RUS	2009
HQ392388	BEL	2009
KY497502	PAK	2010
HQ537518	RUS	2010
HQ537514	RUS	2010
KY497556	PAK	2010
JN706219	THA	2010
JN706208	THA	2010
JN706213	THA	2010
JN706245	THA	2010
KJ751938	SWZ	2010
JN706248	THA	2010
JF719063	IR	2010
JN706235	THA	2010
JN706252	THA	2010
JN706205	THA	2010
LC018668	TUR	2011
KF018787	RUS	2011
KF723266	IND	2011
JN706271	THA	2011
JN706270	THA	2011

KX638540	IND	2011
JN706275	THA	2011
JN706291	THA	2011
KX638547	IND	2012
KX638541	IND	2012
KX638545	IND	2012
KX363088	VNM	2012
KY616899	JPN	2013
MF168067	USA	2013
KX638551	IND	2013
LC172272	JPN	2013
KX363298	VNM	2013
KX638553	IND	2013
KF723268	IND	2013
KP793024	IND	2014
LC028930	JPN	2014
MK050127	IR	2015
MH712903	IND	2015
MK050129	IR	2015
MK829345	IND	2015
MK050128	IR	2015
MH712907	IND	2016
MH712904	IND	2016
MH182444	PAK	2016
MK050137	IR	2016
AB081774	JPN	1987
AB081791	JPN	1988
AB081779	JPN	1988
AB081777	JPN	1988
AB081776	JPN	1988
AB081775	JPN	1988
AB081798	JPN	1989
AB081797	JPN	1989
AB081788	JPN	1989
AB081786	JPN	1989
AB081785	JPN	1989
AB081783	JPN	1989
AB081790	JPN	1990
KT695109	USA	1991
KT695131	USA	1991
D17723	JPN	1992
D17720	JPN	1992
D17718	JPN	1992
DQ886957	IND	1992

EF690753	BGD	1993
AF043681	AUS	1994
AF043680	AUS	1994
AF043679	AUS	1994
AF043682	AUS	1995
AF043684	AUS	1995
AF043683	AUS	1995
AF260938	CHN	1996
AF260937	CHN	1996
HM998612	BRA	1996
HM998613	BRA	1996
JN232040	BRA	1996
JX458959	ARG	1996
JQ710668	ARG	1996
AF260949	CHN	1997
AF260948	CHN	1997
AF260947	CHN	1997
AF260946	CHN	1997
AF260944	CHN	1997
AF260936	CHN	1997
JN232045	BRA	1997
JN232043	BRA	1997
JN232042	BRA	1997
JN232041	BRA	1997
JF968557	BRA	1997
EF690763	BGD	1997
EF690762	BGD	1997
AF260955	CHN	1997
AF260954	CHN	1997
AF260952	CHN	1997
AF260942	CHN	1998
AF260941	CHN	1998
AF260940	CHN	1998
AF260951	CHN	1998
JN232048	BRA	1998
JN232046	BRA	1998
MG181232	MWI	1998
KJ627155	PRY	1998
DQ015681	PRY	1998
JX458961	ARG	1998
AF480291	URY	1998
JN232051	BRA	1999
EF690713	BGD	1999
KT223453	BEL	1999

JN258791	BEL	1999
MG181254	MWI	1999
MG181243	MWI	1999
KJ627121	PRY	1999
JX458962	ARG	1999
JQ710671	ARG	1999
AF480278	URY	1999
JN232064	BRA	2000
JN232057	BRA	2000
KT223454	BEL	2000
MG181309	MWI	2000
MG181276	MWI	2000
MG181265	MWI	2000
KP753172	ZAF	2000
JQ710672	ARG	2000
JN232066	BRA	2001
EF690737	BGD	2001
JN258844	BEL	2001
MG181936	MWI	2001
MG181331	MWI	2001
DQ512998	CHN	2002
DQ512996	CHN	2002
JN232069	BRA	2002
JN232067	BRA	2002
JF968571	BRA	2002
EF690749	BGD	2002
MG181397	MWI	2002
MG181386	MWI	2002
MG181353	MWI	2002
MG181342	MWI	2002
JN232072	BRA	2003
KT920997	IND	2003
DQ512969	VNM	2003
DQ512970	VNM	2003
JN258879	BEL	2003
MG181408	MWI	2003
JQ710675	ARG	2003
DQ207389	IRL	2003
AB585912	CHN	2004
AB585910	CHN	2004
JN232074	BRA	2004
JF968579	BRA	2004
DQ512981	THA	2004
DQ512980	THA	2004

DQ512979	THA	2004
EF199716	THA	2004
EU839915	BGD	2004
JF490528	AUS	2004
JF490170	AUS	2004
MG181441	MWI	2004
MG181419	MWI	2004
KP752609	ZAF	2004
EF088838	JPN	2005
AB585918	CHN	2005
AB585915	CHN	2005
EU708571	CHN	2005
JF968588	BRA	2005
JF968587	BRA	2005
EU839912	BGD	2005
EU839916	BGD	2005
EU839911	BGD	2005
EU839908	BGD	2005
JF490726	USA	2005
JF490619	USA	2005
JF490608	USA	2005
HQ392034	BEL	2005
HQ392009	BEL	2005
MG181474	MWI	2005
KJ753163	ZAF	2005
KJ752078	ZAF	2005
KJ751976	ZAF	2005
JX458967	ARG	2005
JQ710676	ARG	2005
JX470511	CA	2005
JX470510	CA	2005
AB585919	CHN	2006
JF968591	BRA	2006
JF968589	BRA	2006
GQ229041	IND	2006
GQ229040	IND	2006
FJ948852	IND	2006
FJ948850	IND	2006
FJ948846	IND	2006
FJ948844	IND	2006
FJ948841	IND	2006
FJ948840	IND	2006
FJ948855	IND	2006
FJ948849	IND	2006

FJ948848	IND	2006
FJ948847	IND	2006
FJ948845	IND	2006
FJ948839	IND	2006
FJ948837	IND	2006
FJ948832	IND	2006
EU839914	BGD	2006
EU839913	BGD	2006
EU839910	BGD	2006
JF490923	USA	2006
JF490896	USA	2006
JF490847	USA	2006
JF490368	AUS	2006
HQ392132	BEL	2006
KP752839	ZAF	2006
JQ710677	ARG	2006
AB585922	CHN	2007
AB585921	CHN	2007
AB585916	CHN	2007
MK531588	CHN	2007
EU708573	CHN	2007
EU708570	CHN	2007
GU985249	CHN	2007
GU985248	CHN	2007
GU985245	CHN	2007
GU985240	CHN	2007
GU985239	CHN	2007
GU985238	CHN	2007
GQ229043	IND	2007
GQ229042	IND	2007
JN192115	IND	2007
JN192113	IND	2007
JN192112	IND	2007
JN192110	IND	2007
JN192108	IND	2007
JN192107	IND	2007
JN192105	IND	2007
JN192096	IND	2007
JN192087	IND	2007
JN192075	IND	2007
JN192066	IND	2007
JN192063	IND	2007
JN192062	IND	2007
JN192054	IND	2007

GQ996867	THA	2007
HM773829	USA	2007
HM773763	USA	2007
HQ392431	BEL	2007
HQ392198	BEL	2007
KJ753384	ZAF	2007
HQ230028	JPN	2008
MK531587	CHN	2008
JN192135	IND	2008
JN192131	IND	2008
JN192128	IND	2008
JN192127	IND	2008
JN192124	IND	2008
JN192122	IND	2008
JN192121	IND	2008
JN192119	IND	2008
JN192117	IND	2008
JN192116	IND	2008
GQ996875	THA	2008
GQ996871	THA	2008
GQ996869	THA	2008
GQ996868	THA	2008
GQ996866	THA	2008
KP882252	BGD	2008
KP882241	BGD	2008
HM773785	USA	2008
HQ392437	BEL	2008
HQ392360	BEL	2008
HQ392337	BEL	2008
HQ392326	BEL	2008
HQ392321	BEL	2008
HQ392294	BEL	2008
MG181485	MWI	2008
KJ753123	ZAF	2008
KJ752278	ZAF	2008
KJ752199	ZAF	2008
KJ751905	ZAF	2008
KJ412715	PRY	2008
KP883198	MLI	2008
KP883044	MLI	2008
KP883000	MLI	2008
KP882769	MLI	2008
KP882505	GHA	2008
KP882494	GHA	2008

KP882318	GHA	2008
KU973951	CA	2008
KU973944	CA	2008
GQ433993	IRL	2008
MK531586	CHN	2009
KU360939	BRA	2009
KX638517	IND	2009
KX638515	IND	2009
KX638514	IND	2009
KX638513	IND	2009
KX638511	IND	2009
KX638510	IND	2009
KX638509	IND	2009
GQ996879	THA	2009
GQ996853	THA	2009
MK060164	RUS	2009
GU377174	RUS	2009
JN258953	BEL	2009
JN258941	BEL	2009
JN258928	BEL	2009
JN258917	BEL	2009
JN258908	BEL	2009
HQ392381	BEL	2009
KP752987	ZAF	2009
KJ753451	ZAF	2009
KJ753087	ZAF	2009
KJ412911	PRY	2009
KJ412802	PRY	2009
KP883143	MLI	2009
KP882615	GHA	2009
KP752904	ETH	2009
KU973949	CA	2009
KU973947	CA	2009
KP752653	TGO	2009
KJ752042	SEN	2009
KJ751562	SEN	2009
KJ751740	CMR	2009
KJ752243	ZMB	2009
KJ751828	BFA	2009
KJ870927	COD	2009
MK531585	CHN	2010
KU360942	BRA	2010
KX638537	IND	2010
KX638527	IND	2010

KX638526	IND	2010
KX638525	IND	2010
KX638523	IND	2010
KX638522	IND	2010
KX638521	IND	2010
KX638520	IND	2010
KX638519	IND	2010
KX638518	IND	2010
MN067074	IND	2010
JN706223	THA	2010
JN706220	THA	2010
HQ537526	RUS	2010
JQ713096	RUS	2010
KT223451	BEL	2010
KJ753198	ZAF	2010
AB905462	BTN	2010
AB905460	BTN	2010
AB905458	BTN	2010
AB905456	BTN	2010
KP753217	TGO	2010
KP752676	SWZ	2010
KJ752289	GMB	2010
KJ751718	GMB	2010
MK531576	CHN	2011
KX638532	IND	2011
KX638531	IND	2011
KX638530	IND	2011
KX638529	IND	2011
KX638528	IND	2011
MN067115	IND	2011
KT920689	USA	2011
KT921129	USA	2011
MF184781	USA	2011
KP752758	TGO	2011
KJ753297	ZWE	2011
AB796448	JPN	2012
AB796447	JPN	2012
AB796446	JPN	2012
AB796445	JPN	2012
MK531575	CHN	2012
MN067137	IND	2012
MN067126	IND	2012
MN067085	IND	2012
KF723267	IND	2012

MG181562	MWI	2012
MG181529	MWI	2012
KP007181	PHI	2012
LC172403	JPN	2013
KX638552	IND	2013
KX638535	IND	2013
MK531584	CHN	2013
MK531583	CHN	2013
MN066806	IND	2013
MG181672	MWI	2013
MG181683	MWI	2013
MG816524	CHN	2013
MF168306	USA	2013
KX632347	UGA	2013
LC172421	JPN	2013
KY616903	JPN	2014
LC172450	JPN	2014
LC105423	JPN	2014
LC105358	JPN	2014
LC105245	JPN	2014
LC105203	JPN	2014
MG816522	CHN	2014
MK531582	CHN	2014
MH712900	IND	2014
MH712899	IND	2014
MH712898	IND	2014
KP793025	IND	2014
KP793020	IND	2014
KP793019	IND	2014
MK829381	IND	2014
MK829378	IND	2014
MK829377	IND	2014
MK829374	IND	2014
MG181749	MWI	2014
LC434532	IDN	2014
MG652307	DOM	2014
MG652302	DOM	2014
LC311226	JPN	2015
LC311225	JPN	2015
MK531581	CHN	2015
MH712902	IND	2015
MH712901	IND	2015
MK829352	IND	2015
MK829348	IND	2015

MK829347	IND	2015
MK829346	IND	2015
MK829341	IND	2015
MK829340	IND	2015
MG652312	DOM	2015
MH002240	FRA	2015
MK531580	CHN	2016
MK829386	IND	2016
MK829385	IND	2016
MK829365	IND	2016
MK829360	IND	2016
MK829359	IND	2016
MK829358	IND	2016
MK050138	IR	2016
MK531579	CHN	2017
MK050143	IR	2017
MK050141	IR	2017
LC434539	IDN	2018
LC434538	IDN	2018

Table S2 Geographical and temporal information of strains in lineage I and II

Lineage I		Lineage II		Overlap of time
Country	Time-span	Country	Time-span	
ARG	1990~2002	ARG	1996~2006	1996~2002
AUS	1992~2007	AUS	1994~2006	1994~2006
BEL	2000~2009	BEL	1999~2010	2000~2009
		BFA	2009	
		BGD	1993~2008	
BRA	1998~2005	BRA	1996~2010	1996~2005
		BTN	2010	
CA	2005~2008	CA	2005~2009	2005~2008
CHN	2004	CHN	1996~2017	2004
		CMR	2009	
		COD	2009	
		DOM	2014~2015	
ETH	2009	ETH	2009	2009
		FRA	2015	
		GHA	2008~2009	
		GMB	2010	
		IDN	2014~2018	
IND	2006~2016	IND	1992~2016	2006~2016
IR	2010~2016	IR	2003~2017	
JPN	1974~2014	JPN	1987~2015	
		MLI	2008~2009	
		MWI	1998~2014	
PAK	2010~2016	PHI	2012	
PRY	2002~2003	PRY	1998~2009	2002~2003
RUS	1987~2011	RUS	2009~2010	
		SEN	2009	
SWZ	2010	SWZ	2010	2010
		TGO	2009~2011	
THA	2007~2011	THA	2004~2010	2007~2010
TUR	2011	UGA		
URY	1996	URY	1998~1999	
USA	1974~2013	USA	1991~2013	1991~2013
VNM	2012~2013	VNM	2003	
ZAF	2001~2004	ZAF	2000~2010	2001~2004
		ZMB	2009	
		ZWE	2011	
